# Electrochemical Analysis of Attoliter Water Droplets in Organic Solutions through Partitioning Equilibrium

**DOI:** 10.3390/s23042157

**Published:** 2023-02-14

**Authors:** Hyeongkwon Moon, Jun Hui Park

**Affiliations:** Department of Chemistry, Chungbuk National University, Cheongju 28644, Republic of Korea

**Keywords:** droplet, collision, extraction, single-entity electrochemistry, nanoreactor

## Abstract

Herein, we report the electrochemical monitoring of attoliters of water droplets in an organic medium by the electrolysis of an extracted redox species from the continuous phase upon collisional events on an ultramicroelectrode. To obtain information about a redox-free water droplet in an organic solvent, redox species with certain concentrations need to be contained inside it. The redox species inside the droplet were delivered by a partitioning equilibrium between the organic phase and the water droplets. The mass transfer of the redox species from the surrounding organic phase to the droplet is very fast because of the radial diffusion, which resultantly establishes the equilibrium. Upon the collisional contact between the droplet and the electrode, the extracted redox species in the water droplets were selectively electrolyzed, even though the redox species in the organic continuous phase remained unreacted because of the different solvent environments. The electrolysis of the redox species in the droplets, where the concentration is determined by the equilibrium constant of the redox species in water/oil, can be used to estimate the size of single water droplets in an organic solution.

## 1. Introduction

Single-entity electrochemistry has recently attracted significant interest [1,2,3]. In particular, a single entity has been electrochemically monitored individually to study its characteristics, such as metal nanoparticles [4,5], biomaterials [6], nanobubbles [7], liquid droplets [8], and vesicles [9]. This approach is a powerful tool for investigating information about single entities, such as entity concentration [10,11,12], internal redox concentration [13,14], size distribution [15], surface charge [16,17], polydispersity [18], and diffusion coefficients [19]. Monitoring the amperometric current while establishing single-entity electrochemistry by a collisional contact of droplets on UME has been used to estimate droplet sizes [20,21,22]. For example, precise droplet size information has been obtained by using the complete electrolysis reaction in the droplets with redox species within. Upon droplet collision, the redox species in the droplet are depleted via electrolysis, and the droplet sizes can be estimated from the reaction charges by assuming that the droplet has a spherical shape. Several groups, including ours, studied single emulsions (water-in-oil and oil-in-water droplets) via single-entity electrochemistry using redox species (e.g., ferrocene and ferrocyanide) contained beforehand [23,24,25,26]. Furthermore, the electrochemical synthesis of metal particles using a single aqueous droplet has been extensively developed along with the conventional electrodeposition method [27,28,29].

However, in actual samples, single droplets rarely contain redox species; therefore, the droplet detection principle of using an electrolysis reaction inside the droplet is not applicable. Therefore, detecting redox-free aqueous droplets dispersed in a continuous medium is challenging. There have been a few reports on the direct electrolysis of droplets (e.g., water or oil) using solvent molecules as redox species [30,31]. We have previously attempted to study the properties of water droplets via direct electrolysis of water molecules. However, in our previous study, approximately 0.8% of the water molecules in the droplets reacted because of the drastic pH change caused by the reaction product (i.e., protons) [30]. Therefore, the reacted concentrations of the solvent molecules in the droplets are uncertain and can change depending on the external environment. In general, solvent electrolysis in a droplet cannot fully proceed because of a high concentration of solvent molecules, a loss of electrical contact during electrolysis, or bubble formation [32,33,34]. With an uncertain concentration of the redox species (i.e., solvent molecule) reacting in the droplet, the size of the droplet could not be estimated. Therefore, a fixed concentration of redox species in the droplet is necessary for accurate size measurements.

Partitioning equilibrium, which transfers a chemical species from one phase to another and vice versa, is a promising method for collecting redox species in a single droplet and can be used for the electrochemical characterization of a single droplet. Furthermore, the partitioning equilibrium constant determines the concentration of the redox species in the droplet when the concentration of continuous media is known, which can consequently be used for droplet size measurements [20,35].

Additionally, the partitioning equilibrium of redox species could be used to sensitively investigate aromatic compounds in aqueous solutions by preconcentration in oil droplets. If the redox species are more soluble in the aqueous phase than in the organic phase, the equilibrated redox species in the aqueous droplet can be more concentrated than in the organic phase. As a result, the redox species should be carefully chosen to detect droplets in continuous media while taking solubility in each phase and the equilibrium constant into account. For example, benzene-1,4-diol is preliminarily dissolved in petroleum storage to prevent the oxidation of petroleum, which could be used for detecting water contamination in petroleum tanks.

In this study, we analyzed redox-free water droplets using redox species transferred from an organic medium by a partitioning equilibrium. After the redox species in the organic medium were preliminarily determined by the experimenter, they were transferred to the water droplet until establishing an equilibrium concentration. Mass transfer of the redox species from the surrounding organic phase to the droplet is very fast due to radial diffusion, thereby an equilibrium state is quickly obtained. In this experiment, 4-aminophenol (AP) was used as the redox species. To differentiate the single droplet’s electrochemical response, the electrochemical response from the continuous media should be minimized. The reaction in the organic medium was suppressed by controlling the composition of the electrolyte. Therefore, the droplets containing the equilibrated redox species contact the electrode surface, causing current spikes because of the electrochemistry of a single droplet. By investigating the electrochemistry of AP in the water droplet, the size distribution of the water droplet was determined.

## 2. Materials and Methods

### 2.1. Reagents and Materials

1,2-dichloroethane (DCE, 99.5%), acetone (99.5%), anhydrous alcohol (99.5%), AP (99%), and anhydrous MgSO_4_ (99.5%), were purchased from Samchun Pure Chemicals. Hexafluorophosphate (TBAPF_6_, 99% purity) was purchased from Alfa Aesar. A Pt wire (diameter: 25 μm) was obtained from Goodfellow. Borosilicate capillary tubing (1.5 mm outer diameter × 0.75 mm inner diameter) was obtained from FHC Inc. (Bowdoin, ME, USA). Millipore water (>18 MΩ cm) was used for all the experiments.

### 2.2. Instruments

Electrochemical experiments were performed using a 760E potentiostat (CH Instruments, Austin, TX, USA) with a three-electrode cell placed in a Faraday cage. A 25 μm Pt-UME was used as a working electrode. Ag/AgCl (in saturated KCl) and Ag/Ag^+^, 10 mM AgNO_3_ (in acetonitrile) reference electrodes were chosen for the aqueous and organic solvent phases, respectively, whereas a Pt wire (1 mm diameter) was used as the counter electrode. To analyze the particle size, dynamic light scattering (DLS, NanoBrook 90Plus, Brookhaven Instruments Corporation, Holtsville, NY, USA) was used. A microtip-equipped horn sonicator (Q700 ultrasonic processor, Qsonica, Newtown, CT, USA) was used to create the water-in-oil emulsions. Before all the experiments, the working electrode was rinsed with acetone, ethanol, and water. 

### 2.3. Preparation of Water-In-Oil Emulsions (Water Droplets)

Water-in-oil emulsions were prepared by washing DCE with water and 100 mM MgSO_4_ in distilled water. To make a 5200 μL emulsion solution, an aqueous solution (200 μL) was added to the DCE solution (5 mL), and the mixture was vortexed for at least 30 s. Using pulse mode (5 s on/5 s off, six cycles), ultrasonic power (700 W, amplitude 30%) was applied after vortexing the mixture. The DLS revealed that the average droplet diameter was 716.1 nm. After 100 s of emulsion injection, electrochemical measurements were performed to achieve partition equilibrium in the AP. 

### 2.4. Preparation of the Pt-UME

The UME was prepared according to the procedure developed in our laboratory [35]. A 25 µm Pt wire (Goodfellow, Huntingdon, UK) was briefly sealed in a borosilicate glass capillary (1.5 mm O.D. × 0.75 mm I.D. Sutter), which was sonicated in hexane, toluene, IPA, ethanol, and water. The electrode was then polished with SiC abrasive sandpaper (400, 1000, 1200, 2000, and 2500 grit; Buehler, Lake Bluff, IL, USA) until a mirror-like surface was observed. The electrode surface area was determined using FeMeOH as the redox mediator. Before each electrochemical experiment, all UMEs were polished using 4000-grit SiC sandpaper (R&B Co., Ltd., Daejeon, Republic of Korea).

## 3. Results and Discussion

Biphasic redox species, which could be transferred to the water droplets by the partitioning equilibrium, were carefully selected to detect redox-free water droplets in the organic phase. An ideal biphasic soluble redox species should exhibit a large difference in solubility between the organic and aqueous phases. Hence, the redox species should have high water/organic partition coefficients, leading to a high concentration of the species in the aqueous phase. Under these conditions, the redox species in the water droplets can be established at a high fixed concentration.

**Scheme 1:**
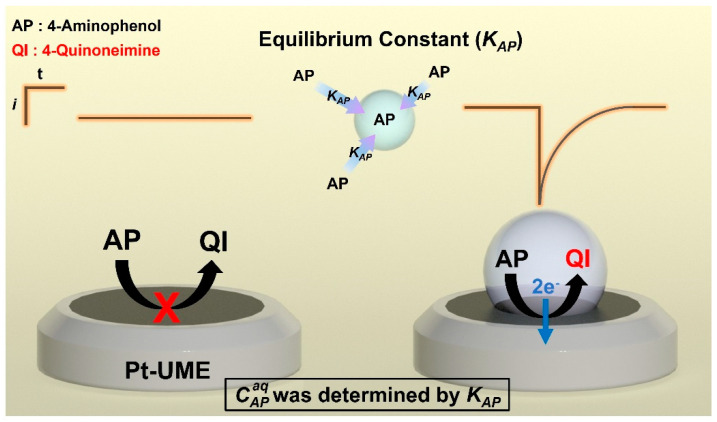
Schematic illustration of the partitioning equilibrium of AP from the DCE phase to a single water droplet. Under oxidative potential on Pt-UME, the electrochemical response of the AP within the water droplets was monitored.

**Scheme 2:**
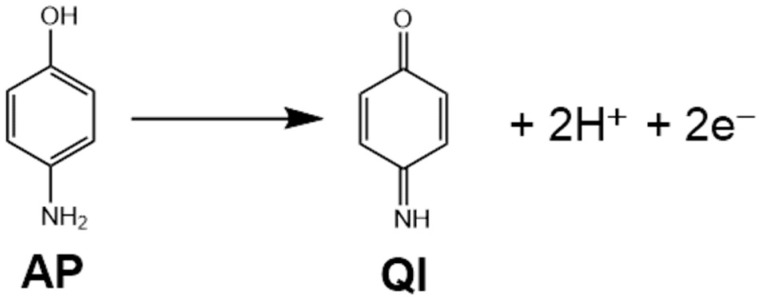
Schematic illustration of electrochemical oxidation of 4-aminophenol (AP) to 4-quinoneimine (QI).

As a model system, AP, with solubilities of 15 g/L in water and 0.40 g/L in DCE at 25 °C, was chosen as a redox probe that can be dissolved in both the water and DCE phases (Figure S1). Owing to the large difference in solubility between the water and DCE phases, AP dissolved in DCE can be successfully concentrated into the water droplet. Attoliter droplets dispersed in the DCE phase can quickly establish a partition equilibrium (*K_AP_*) owing to the random movement of the droplets and the radial diffusion of AP. Compared with the AP concentration in the continuous organic phase of DCE, the water droplets achieved relatively high AP concentrations owing to the partitioning equilibrium. As shown in Scheme 1, the random movement of water droplets in continuous media can cause an electrochemical response when they collide on the Pt-UME. When the collided water droplet is adsorbed on Pt-UME, AP in the water droplet is instantly oxidized to 4-quinoneimine (QI), showing a transient current increase and rapid decay owing to the depletion of AP within the water droplet. However, in this system, the same redox species coexist in the organic phase. Therefore, to monitor the collision of droplets, the oxidation of AP in the organic phase should be controlled. The redox reaction was noted to have different oxidation potentials between the organic and aqueous solvents [35]. The solvation energy of redox species and the ohmic drop induced the differences in oxidation potential for AP depending on the solvent system. In addition, we intentionally decreased the ionic strength of the DCE phase to increase the ohmic overpotential for AP oxidation. Therefore, the oxidation of AP in DCE was effectively suppressed, and the current from it was negligible. The oxidation reaction of AP in an aqueous solution is shown in Scheme 2.

**Figure 1:**
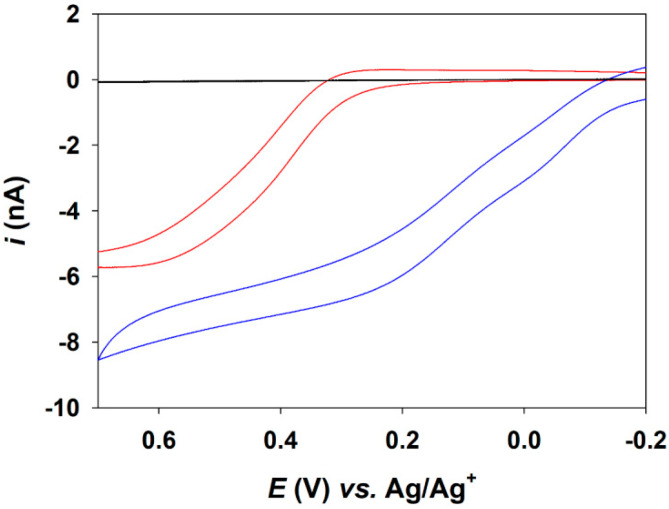
Cyclic voltammetry of 1 mM AP in DCE (black), 1 mM AP in DCE containing 100 mM TBAPF_6_ (red), and 1 mM AP in an aqueous solution containing 100 mM MgSO_4_ (blue). A 25 um Pt-UME was used, and the scan rate was 100 mV/s.

Cyclic voltammetry (CV) was conducted to investigate the electrochemical properties of AP in different solvent systems, including DCE solutions containing 1 mM AP with and without 100 mM TBAPF_6_, and a water solution containing 1 mM AP and 100 mM MgSO_4_. In the absence of a TBAPF_6_-supporting electrolyte in DCE, AP was barely oxidized until a potential of +0.7 V, as shown in Figure 1. In the presence of 100 mM TBAPF_6_ in DCE, however, AP was oxidized at a potential of over +0.2 V. Conversely, the electrochemical oxidation of AP in the aqueous solution started at −0.2 V vs. Ag/Ag^+^. The oxidation potential of AP in the two electrolyte solutions was compared, and the AP in the aqueous solution was oxidized at a potential approximately 0.4 V more negative than that in the DCE solution containing TBAPF_6_. This was mainly affected by the solvation energy difference of AP in the electrolyte-solvent system. Additionally, by removing the supporting electrolyte in the DCE phase (i.e., TBAPF_6_), which increases the solution resistance, the oxidation potential of AP in the DCE phase shifted to a more positive value due to the ohmic potential drop. Therefore, the difference in the oxidation potential can be used to selectively oxidize AP within the water droplet.

**Figure 2:**
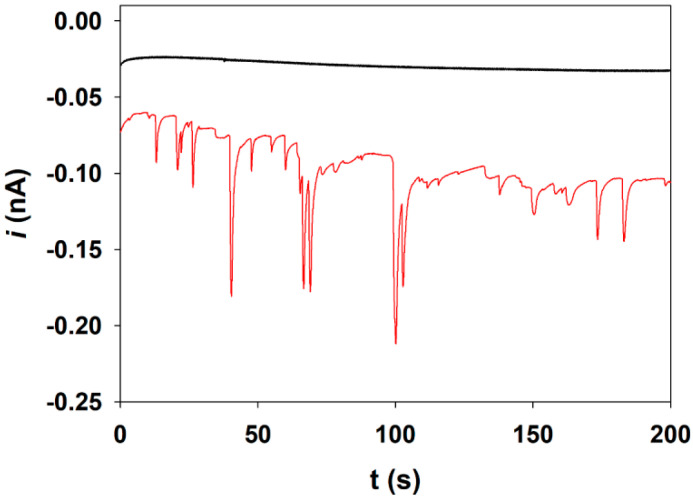
Amperometric *i-t* curves were monitored for investigating the single water droplets in 3 mM AP DCE (black) and 3 mM AP DCE in the presence of a water droplet (red), using a 25 μm Pt-UME at +0.6 V (vs. Ag/Ag^+^).

A constant potential of +0.6 V was applied to the Pt-UME to monitor a single collision event of a water droplet. As shown in Figure 2, there was no detectable current peak in DCE containing 3 mM AP in the absence of water droplets (black line). However, a spike-shaped current was observed in the presence of water droplets (100 mM MgSO_4_) in a DCE solution containing 3 mM AP. The spiky current response corresponds to the electrolysis of AP in a water droplet. The spiky shape showed an instantaneous increase and rapid decay, implying that the electrolysis reaction started immediately at the collisional contact and rapidly attenuated due to the depletion of the AP in the droplet. The AP in the water droplet was transferred from the DCE phase by a partition equilibrium. Assuming that the colliding droplet has already established equilibrium, the concentration of AP inside the droplet can be easily obtained using the equilibrium constant of AP in the DCE/water solution. The equilibrium constant could be obtained electrochemically from the experiment of a solution in contact with an immiscible electrolyte solution, as shown in the following paragraph. The equilibrium concentration of AP in a water droplet can be used to estimate the droplet size by relating the integrated charge of the single spiky current.

**Figure 3:**
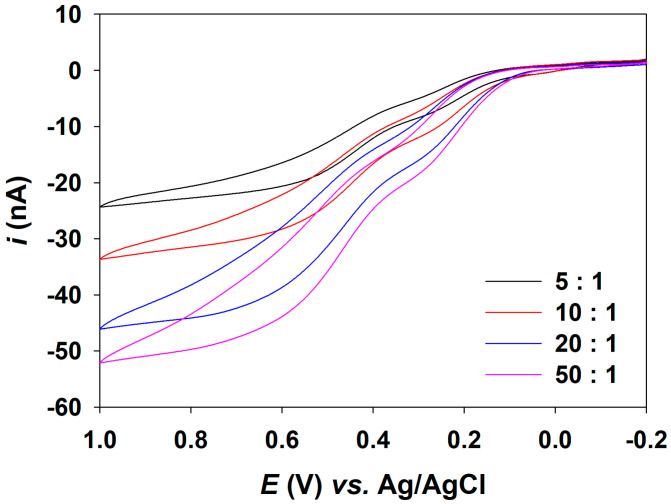
Cyclic voltammograms were measured at the aqueous part of the mixture of a DCE solution containing 1 mM AP and an aqueous solution containing 100 mM MgSO_4_ solution with various volume ratios (5:1, 10:1, 20:1, and 50:1, respectively), using a 25 μm Pt-UME at a scan rate of 100 mV/s.

To determine the equilibrium constant of AP, 100 mM MgSO_4_ aqueous solution and 1 mM AP DCE solution were mixed at various volume ratios and gently stirred to reach a steady state. CV was performed in the aqueous part of the stabilized mixed solution using the Pt-UMEs. As shown in Figure 3, the CV curves of AP in the aqueous phase were obtained at various DCE solution/aqueous solution ratios, and the AP concentrations were determined using the following equation:

(1) $$C_{AP,aq}=\frac{i_{lim}}{4nFD_{AP}r_{UME}}$$

where $i_{lim}$ is the limiting current, n is the number of electrons in the redox reaction (for AP, n = 2), $D_{AP}$ is the diffusion coefficient of AP in water at 25 °C, and $r_{UME}$ is the radius of Pt-UME. The steady-state concentration of AP in the DCE solution was calculated using the following equation:

(2) $$C_{AP,DCE}=\frac{C^{*}\times V_{DCE}-C_{AP,aq}\times V_{aq}}{V_{DCE}}$$

where $C^{*}$ is the initial AP concentration in DCE, $C_{AP,aq}$ is the AP concentration in the aqueous phase at equilibrium, and the volumes of the phases are $V_{DCE}$ and $V_{aq}$, respectively. The equilibrium constants ($K_{AP}$) were obtained by substituting Equations (1) and (2) into Equation (3).

(3) KAP=CAP,aqCAP,DCE

Table 1 presents the experimentally obtained equilibrium constants of AP in solutions with various volume ratios of 1 mM AP in DCE and 100 mM MgSO_4_ in water. The average value of $K_{AP}$ was 6.88. 

The equilibrium concentration of AP in a water droplet was calculated using Equation (4).


(4) $$C_{AP,drop}=C^{*}\times \frac{V_{DCE}K_{AP}}{V_{DCE}+V_{aq}K_{AP}}$$

(5) $$C_{AP,drop}=C^{*}\times K_{AP}\;\;(\mathrm{when}\;V_{DCE}\gg V_{aq}K_{AP})$$

From Equation (4), the concentration of AP within the water droplets was determined to be 20.1 in the 3 mM AP DCE solution. Because the water content was less than 0.4% in the real samples, the concentration of AP in the DCE phase was minimally affected by partitioning into the aqueous phase, which had a very small volume. Therefore, we can simplify Equation (4) to Equation (5) because $V_{DCE}$ is significantly greater than $V_{aq}K_{AP}$. The concentration of AP in a droplet can be easily calculated using Equation (5). Based on complete electrolysis inside the droplet during collision, the diameter of the droplet can be calculated using Equation (6).

(6) $$d_{drop}=\sqrt[{3}]{\frac{6Q}{n\pi FC_{AP,drop}\;}}$$

here $d_{drop}$ is the diameter of the water droplet, Q is the integrated charge from single collision peaks, n is the number of electrons, and $C_{AP,drop}$ is the initial concentration of the redox species in the DCE.

**Figure 4:**
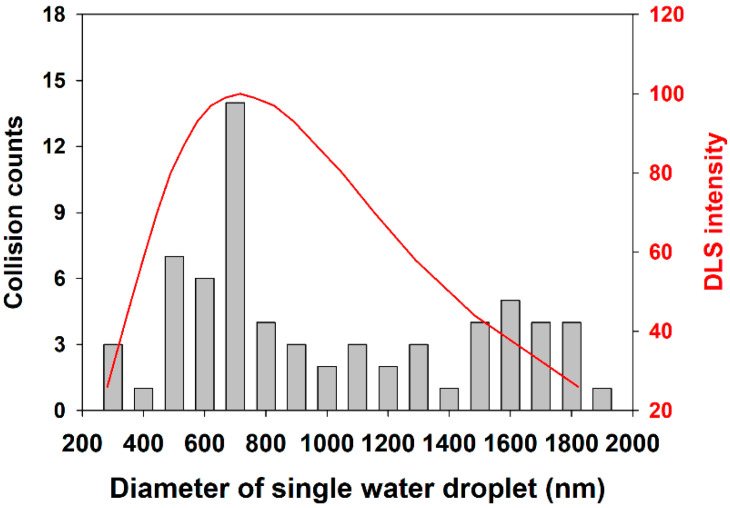
The size distribution of water droplets was obtained from the collision method (bars) and DLS measurements (red line).

The size distribution of the water droplets based on Equation (6) is shown in Figure 4. The droplet size distributions obtained using the single-droplet electrochemistry method (bars) are consistent with the values obtained from the DLS measurements (red line), implying that the AP concentration estimated from the electrochemical experiment of a solution in contact with an immiscible electrolyte solution is reasonable. Moreover, some large droplets (>1800 nm) that were not detected by the DLS measurements were observed using the electrochemical method. This is consistent with previous studies that have shown that electrochemical single-particle analysis is advantageous for detecting large droplets. Therefore, the size of an aqueous droplet can be precisely measured via the electrochemistry of a single entity using an extracted redox species.

## 4. Conclusions

We established a novel electrochemical detection method for redox-free water droplets in an organic medium based on the oxidation of AP, extracted from an organic phase, in the water droplet. Water droplets in organic media, such as DCE, can attain an equilibrium concentration of AP by phase transfer. At an optimized electrode potential, AP was selectively electrolyzed only in the aqueous phase because of the solvation energy difference between the aqueous and organic phases, whereas AP in DCE was not electrolyzed at the same potential. Therefore, spiky current responses were obtained owing to the electrolysis of AP inside the droplet when the water droplets collided with the electrode surfaces. The droplet size was calculated by integrating the charges of collisional current spikes and assuming that the AP concentration inside the droplet is fixed. The concentration of AP injected into the water droplet was estimated from the equilibrium constant of AP between DCE and water that was measured electrochemically in the DCE/water immiscible electrolyte solution at equilibrium. The electrochemically obtained droplet sizes corresponded well with the DLS data. Therefore, we introduced a novel size measurement system for redox-free water droplets in organic media.

## Figures and Tables

**Table 1:** Electrochemically obtained equilibrium constant ($K_{AP}$) of AP.

$\mathrm{Ratio}\;(V_{DCE}:V_{aq})$	$C_{AP,\;\;\;DCE}^{*}\;(mM)$	$C_{AP,\;aq}\;(mM)$	$C_{AP,\;DCE}\;(mM)$	$K_{AP}$
5:1	1	2.85	0.43	6.63
10:1		3.94	0.61	6.50
20:1		5.40	0.73	7.40
50:1		6.14	0.88	7.00

## Data Availability

Not applicable.

## Supplementary Materials

The flowing supporting information can be downloaded at: https://www.mdpi.com/article/10.3390/s23042157/s1, Figure S1: Electrochemical data for determining concentration of 4-aminophenol, Figure S2: Additional i-t curve data of collision events of water droplet, and Figure S3: *i-t* curve data with an injection of water droplets during chronoamperometry.
