# Supplementary material for: Electrochemical Analysis of Attoliter Water Droplets in Organic Solutions through Partitioning Equilibrium
Source: Sensors (Basel). 2023 Feb 14;23(4):2157. doi: 10.3390/s23042157 (PMC9959340; doi:10.3390/s23042157)
Supplement: Supplementary file 1 [file sensors-23-02157-s001.zip › sensors-2190637-supplementary.pdf]

Supporting Information

Electrochemical analysis of attoliter water droplets through organic solution by partitioning equilibrium

*Hyeongkwon Moon and Jun Hui Park**

Department of Chemistry, Chungbuk National University, Cheongju 28644, Republic of Korea

AUTHOR INFORMATION

Corresponding Author

E-mail: JHP@chungbuk.ac.kr; Tel: +82-43-261-2287; Fax: +82-43-267-2279

Table of Contents

1. Electrochemical data for determining concentration of 4-aminophenol…….…………….……...…S1
2. Additional i-t curve data of collision events of water droplet…………………………………….S2

3. *i-t* curve data with an injection of water droplets during chronoamperometry.…………………..S3

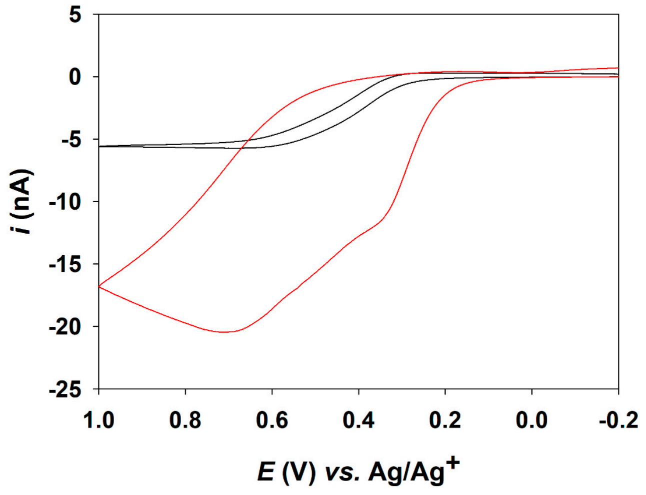

Figure S1. Cyclic voltammetry of 1 mM AP in DCE solution containing 100 mM $TBAPF_6$ (black) and AP saturated DCE solution containing 100 mM $TBAPF_6$ (red). Saturated concentration of AP in DCE solution was estimated using Eq. S1.

$$i_{lim} = 4nD_{AP}r_{UME}C_{AP} \qquad \text{(S1)}$$

Here, i_{lim} is limiting current, n is the number of electrons in the redox reaction, D_{AP} is diffusion coefficient of AP, r_{UME} is the radius of Pt-UME, and C_{AP} is concentration of AP. The calculated saturated concentration of AP in DCE solution was ca. 3.64 mM at 25ºC.

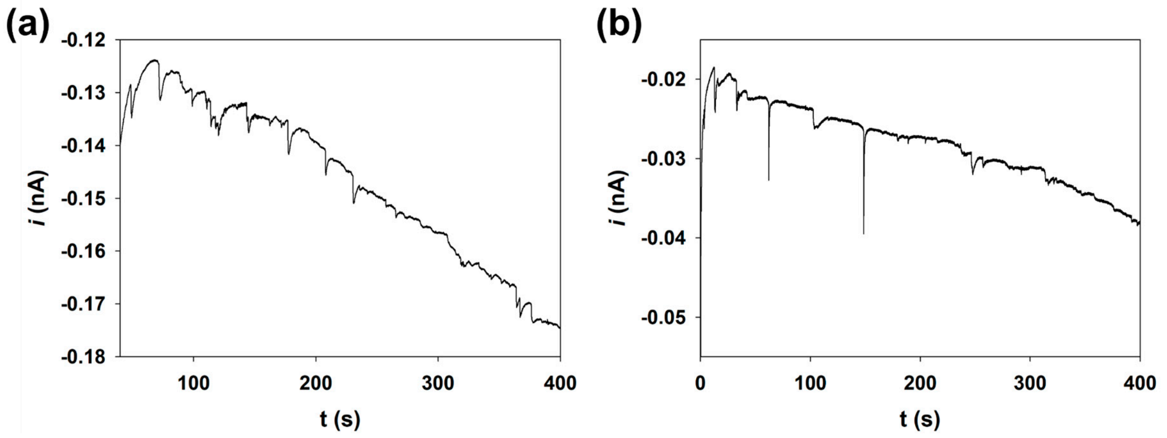

Figure S2 (a), (b) *i-t* curves measured for monitoring the collision of the single water droplets (100 mM $MgSO_4$) using 25 μm Pt UME at +0.6 V *vs.* Ag/Ag^+ in DCE containing 3 mM AP.

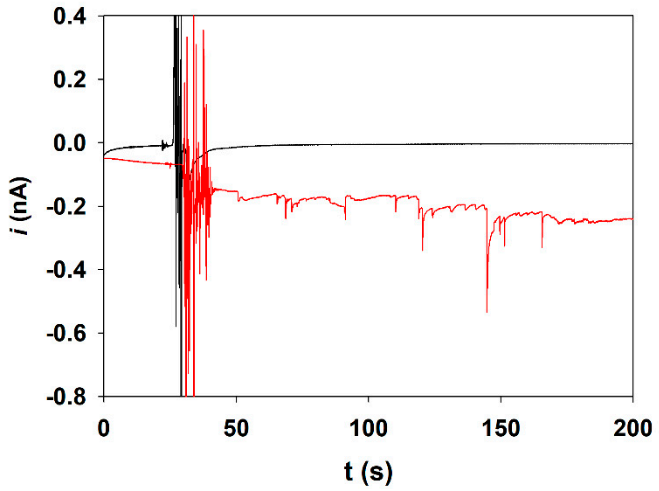

Figure S3. Amperometric *i-t* curve recorded using 25 μm Pt-UME when water droplets were injected at 30s into the the pure DCE solution (black) and DCE solution containing 3 mM AP (red). The large noise signal at 30 s were caused by the injection process of water droplets (e.g., opening of the Faraday cage).
